# Bystander communication and cell cycle decisions after DNA damage

**DOI:** 10.3389/fgene.2015.00063

**Published:** 2015-02-27

**Authors:** Himjyot Jaiswal, Arne Lindqvist

**Affiliations:** Department of Cell and Molecular Biology, Karolinska Institutet, Stockholm, Sweden

**Keywords:** bystander effect, p53, cell cycle, senescence, DNA damage, cell cycle exit, G2

## Abstract

The DNA damage response (DDR) has two main goals, to repair the damaged DNA and to communicate the presence of damaged DNA. This communication allows the adaptation of cellular behavior to minimize the risk associated with DNA damage. In particular, cell cycle progression must be adapted after a DNA-damaging insult, and cells either pause or terminally exit the cell cycle during a DDR. As cells can accumulate mutations after a DDR due to error-prone DNA repair, terminal cell cycle exit may prevent malignant transformation. The tumor suppressor p53 plays a key role in promoting terminal cell cycle exit. Interestingly, p53 has been implicated in communication of a stress response to surrounding cells, known as the bystander response. Recently, surrounding cells have also been shown to affect the damaged cell, suggesting the presence of intercellular feedback loops. How such feedback may affect terminal cell cycle exit remains unclear, but its presence calls for caution in evaluating cellular outcome without controlling the cellular surrounding. In addition, such feedback may contribute to how the cellular environment affects malignant transformation after DNA damage.

## INTRODUCTION

Changes in the genome can be a potential threat to the cell and to organism survival. However, the genome is continuously exposed to a variety of genotoxic stresses. These are endogenous insults such as the production of reactive oxygen species (ROS) or various metabolite byproducts, or exogenous insults such as UV radiation, heavy metals, air pollutants, bacterial toxins, and inflammatory responses. All of these agents cause structural damage and can hinder or abolish cellular processes as transcription or DNA replication. Of the various DNA lesions, DNA double strand breaks (DSBs) are considered most deleterious, because if unrepaired they can lead to chromosomal aberrations such as deletions, translocations, and amplifications. These chromosomal aberrations may result in deregulation of gene expression and altered cellular function, which may eventually cause cell death or tumor initiation and progression (Lord and Ashworth, 2012).

To minimize the risk to genome integrity, cells have evolved the DNA damage response (DDR)—a highly regulated signaling network that responds to the presence of DNA lesions (Bartek and Lukas, 2007; Jackson and Bartek, 2009). A prime function of the DDR is to ensure that lesions in DNA are recognized and repaired. Simultaneously, the repair needs to be coordinated with other cellular processes, in particular cell cycle progression. Therefore, the DDR can be divided into two major pathways, one that assembles and repairs the lesions and one that amplifies and conveys the signal away from the break site to modify cellular behavior. In all eukaryotes these two processes are initiated by sensor proteins such as the Mre11-Rad50-Nbs1 (MRN) complex or the Ku70/Ku80 dimer, that detect the presence of DSBs. The binding of sensor proteins to damaged DNA recruits the phosphoinositide 3-kinase related kinases ATM, ATR, or DNA-PK leading to activation of these kinases (Falck et al., 2005). Once activated these kinases initiate cascades that enforce local and global rearrangement of chromatin, involving recruitment of multiple proteins and posttranslational modifications as phosphorylation, ubiquitylation, sumoylation, and methylation (Lukas et al., 2011). For example, phosphorylation of histone 2A variant (H2AX) at C-termini near a break site by ATM serves as a platform for the protein MDC1, who in turn can function as a recruitment platform for the ubiquitin ligase RNF8. RNF8-mediated ubiquitylation recruits RNF168, whose ubiquitylation of chromatin proteins attract BRCA1 and 53BP1, proteins that affect how the DNA break will be repaired (Kolas et al., 2007; Bekker-Jensen and Mailand, 2011).

## DSB REPAIR IS NOT ALWAYS PERFECT

A majority of DSBs are repaired by three pathways—homologous recombination (HR), non-homologous end joining (NHEJ), and microhomology-mediated end joining (MMEJ). NHEJ is a fast repair process using template independent ligation of two ends of DNA and is functional throughout the cell cycle; in contrast HR is a slow repair process that depends upon the use of a sister chromatid as template and is functional only in late S- and G2 phase of the cell cycle (Chapman et al., 2012). Whereas NHEJ only requires minor modifications of the DNA to allow for ligation, HR requires resection to create stretches of single-stranded DNA that can be used for base-pairing with the sister chromatid (Hartlerode and Scully, 2009). The amount of resection at a DSB is influenced by proteins as BRCA1 and 53BP1, and is considered as a determinant for which repair mechanism will be utilized (Chapman et al., 2012). Importantly, during NHEJ the DNA ends are frequently modified to allow efficient ligation, resulting in a change in the genetic information (Lieber, 2010). Similarly, MMEJ, an alternative version of end-joining contains even larger modifications of DNA ends, giving rise to deletions (Decottignies, 2013). In contrast, due to the use of a template for sequence information, HR is largely considered as an error-free repair process, although its accuracy is debated. Two different processes are described to support that HR may be an error-prone process—first the unequal sister chromatid exchange (SCE) which has been observed in highly repetitive sequences, and second the involvement of translesion synthesis polymerases in synthesizing the DNA (Guirouilh-Barbat et al., 2014). Thus, cells that have repaired DSBs are likely to contain changes in the genetic information.

## CHECKPOINT MAINTENANCE IS NOT ALWAYS PERFECT

The repair of damaged DNA needs to be coordinated with various other cellular processes, in particular cell cycle progression. Therefore, in addition to stimulating repair, the DDR enforces a cell-cycle arrest, referred to as a DNA damage checkpoint. At the heart of the checkpoint are the ATM and ATR kinases, which initiate a signaling cascade by phosphorylating the effector kinases Chk2 and Chk1. Chk1/Chk2 in return phosphorylate cell cycle regulators as Cdc25 phosphatases. Phosphorylation of Cdc25s leads to their functional inactivation and subsequent inhibition of Cdk activity, causing rapid inhibition of cell cycle progression (Peng et al., 1997; Mailand et al., 2000; Karlsson-Rosenthal and Millar, 2006). In addition, inhibition of indirect regulators of Cdk activity as Plk1 and Aurora A support a rapid cell cycle arrest (Smits et al., 2000; Krystyniak et al., 2006). Checkpoint signaling also maintains the arrest by stabilizing p53 that transcriptionally regulates a large number of genes involved in DDR and other stress pathways (Allen et al., 2014). In addition, p38-dependent pathways contribute to regulate protein expression to maintain a checkpoint over time (Reinhardt et al., 2010). However, although the DDR is a very tightly regulated process, evidence of H2AX phosphorylation, chromosomal rearrangements and breakage during the transition from G2 to mitosis suggest that checkpoint signaling is not always stringent (Syljuasen et al., 2006; Deckbar et al., 2007; Lobrich and Jeggo, 2007). Thus, cells that have initiated a DDR and a checkpoint arrest may resume proliferation before all damaged DNA is repaired.

## CELL CYCLE EXIT

As an alternative to a temporal cell cycle arrest, cells may permanently leave the cell cycle and become senescent. The duration from infliction of DNA damage to cell cycle exit depends directly on the cell cycle state. Whereas an untransformed G2 cell exits the cell cycle if damage is not repaired within a couple of hours, an S-phase cell first finishes DNA replication and only leaves the cell cycle in G2 (Baus et al., 2003; Krenning et al., 2014; Müllers et al., 2014). Cell cycle exit in G2 phase depends on activation of the ubiquitin ligase APC/C-Cdh1, which efficiently targets a large amount of cell cycle regulators for proteasome-mediated degradation (Wiebusch and Hagemeier, 2010). How APC/C-Cdh1 is activated after DNA damage remains unclear, but the process depends on expression of p53 and its transcriptional target p21, and at least in the case of Cyclin B1, nuclear translocation of the protein to be degraded (Wiebusch and Hagemeier, 2010; Johmura et al., 2014; Krenning et al., 2014; Müllers et al., 2014). Thus, the regulation of p53 is a key determinant for whether cell cycle exit or resumed proliferation occurs after initiation of a DDR.

## p53 AND CELL FATE

The level and activity of p53 is upregulated in response to various stresses and has been shown to play a role in different pathways including DDR, hypoxia, apoptosis, metabolism and senescence (Gonfloni et al., 2014; Pflaum et al., 2014). Functioning as a complex signaling node, the p53 protein contains a large amount of post-translational modifications, which together with differential affinity for transcriptional elements and expression of regulatory proteins impact on cell fate decisions (Kruse and Gu, 2009; Carvajal and Manfredi, 2013). Interestingly, although p53 levels are similarly induced, different stimuli can elicit different responses on p53-transcription targets such as p21 (Espinosa et al., 2003; Donner et al., 2007), highlighting that p53 function may be modulated by the integration of a wide variety of signaling pathways (Sullivan et al., 2012). One factor that can affect p53 function is its temporal dynamics in cells. Rather than accumulating at a certain level, cellular p53 can oscillate after induction of DSBs (Lahav et al., 2004). In contrast to sustained p53 induction that stimulates cell cycle exit, the oscillatory pulses of p53 favor eventual resumption of proliferation after damage (Purvis et al., 2012). However, exactly how integration of signals determines p53 behavior remains unclear, in particular in the context of a population of cells.

## THE BYSTANDER RESPONSE

During the past few decades the DDR pathway has been studied extensively in cells that have experienced damage directly. However, cells experiencing a DDR can communicate this to surrounding cells (Klammer et al., 2013). The first evidence of propagation of the DDR came from experiments performed in Chinese hamster ovary cell lines, in which 1% of nuclei hit by α-particles resulted in more than 30% of the cell population showing increased incidence of SCE (Nagasawa and Little, 1992). Supported by other observations, this phenomenon was later termed the radiation-induced bystander effect (RIBE), which is defined as physiological changes in unirradiated cells manifested by cells exposed to radiation (Sokolov et al., 2005, 2007; Klammer et al., 2013). Apart from SCE, various biological consequences of RIBE have been observed in different studies such as genomic instability, micronuclei formation, apoptosis, micro RNA (miRNA) regulation, and differentiation (Lorimore et al., 1998; Belyakov et al., 2002; Kovalchuk et al., 2010; Vinnikov et al., 2012). A common feature of RIBE seems to be induction of DNA damage. Indeed, Ku70, Ku80, or DNA-PKcs knockout bystander cells that are repair deficient are sensitive to the induction of mutations and chromosomal aberrations (Little et al., 2003; Nagasawa et al., 2003). However, the number of DSBs generated in directly irradiated and bystander cells differ, and point mutations are predominant in bystander cells as compared to partial or total gene deletion in directly irradiated cells (Little et al., 1997; Huo et al., 2001; Sedelnikova et al., 2007). Mechanistically, deregulation of redox homeostasis may be a major cause of DNA damage in bystander cells (Azzam et al., 2002; Sokolov et al., 2007). Indeed, addition of Vitamin C or E to cell culture reduces the frequency of micronuclei formation, suggesting that ROS contributes to DNA damage formation (Narayanan et al., 1997; Konopacka and Rzeszowska-Wolny, 2006). The occurrence of DSBs in bystander cells is more frequent during DNA replication or active transcription, indicating that energy-dependent processes may underlie some of the damage (Burdak-Rothkamm et al., 2007; Dickey et al., 2012). In addition, these processes involve opening up double-stranded DNA, suggesting a mechanism for how ROS-induced single-stranded breaks can be transformed to DSBs, and indicating that the bystander effect may be particularly efficient during late cell cycle stages where replication and transcription is high.

The bystander effects appear to be cell and genotype specific and also depend upon the type of radiation (Baskar, 2010). Most of the RIBE studies have been performed in cell and tissue culture models where non-irradiated cells were co-cultured with either irradiated cells or with the conditioned medium from irradiated cells. Using mice models, Koturbash et al. (2008) showed that the bystander effect occurs *in vivo* as cranial irradiation led to DNA damage in protected spleen tissues. The RIBE also led to a profound epigenetic change in different bystander parts of the animal and, interestingly, the bystander response could differ between male and female (Besplug et al., 2005; Koturbash et al., 2006, 2007).

The above observations suggest that paracrine or endocrine signaling molecules from irradiated cells are responsible for the bystander effect. However, in addition to secretion of extracellular factors, transmission through gap junctions has also been implicated in RIBE, suggesting that multiple factors may propagate a bystander effect (Azzam et al., 2001; Hubackova et al., 2012; Klammer et al., 2013; He et al., 2014). Some of the factors implicated in transmitting the bystander response are interleukins, transforming growth factor beta (TGFβ), and nitric oxide (NO) (Iyer et al., 2000; Shao et al., 2002; Dieriks et al., 2010). As a consequence of RIBE, a DNA damage-response pathway is initiated in bystander cells. Apart from the p53 pathway, the DDR also initiates stress signaling through JNK and p38 MAPK signaling cascades including NF-kB, a major regulator of cell survival, inflammation, autophagy, and differentiation (Azzam et al., 1998; Piret et al., 1999). Activation of such a signaling network reprograms a cell to react to external danger and may coordinate a response in a complex tissue environment.

## RECIPROCAL BYSTANDER EFFECT

Proper tissue homeostasis is dependent on bidirectional rather than unidirectional communication between cells. It is therefore reasonable to expect that an exchange of signaling molecules between non-irradiated and irradiated cells occurs (Goldberg and Lehnert, 2002; Chen et al., 2011; Widel et al., 2012; He et al., 2014). Indeed, the first observation of bidirectional communication between cells was seen by Mackonis et al. (2007), who reported an increased rate of survival of cells receiving a high radiation dose when their nearby cells received a low radiation dose. This interesting observation was termed a type III effect. Later on Chen et al. (2011) showed that there is a decrease in micronuclei formation and apoptosis in irradiated cells when co-cultured with non-irradiated cells. However, although the mechanisms of a reciprocal bystander effect are not yet clear, recently He et al. (2014) used co-culture of irradiated macrophages and non-irradiated hepatocytes to postulate that cAMP released from bystander hepatocytes could lead to a decreased micronuclei formation in irradiated macrophages. These studies suggest that reciprocal communication is important to react to external damage in an efficient and flexible manner. Interestingly, incorporation of both bystander and reciprocal bystander responses suggests the presence of intercellular feedback loops that may augment responses in both damaged and non-damaged cells.

## p53 IN THE BYSTANDER RESPONSE

One of the promising candidates that can function as a connecting link between intrinsic and extrinsic signals is the p53 protein. Apart from cell autonomous responses, such as activation in response to DSBs in bystander cells, p53 also plays a role in transmission of the bystander response (Lorimore et al., 2013). In particular, cytochrome C release from damaged cells has been shown to be involved in RIBE in a p53-dependent manner, suggesting that p53 can both transmit and respond to RIBE (He et al., 2011). The oscillatory behavior of p53 over time has attracted the attention of modeling efforts to predict the potential outcome on cell fate (Lev Bar-Or et al., 2000; Geva-Zatorsky et al., 2006; Wee et al., 2009). A recent study based on mathematical modeling proposed that cytochrome C could couple the p53 oscillatory behavior in damaged and non-damaged cells to enhance the robustness and sustainability of p53 pulses (Kim and Jackson, 2013). Although this model needs further validation in an experimental setup, a reciprocal bystander effect imposed by cytochrome C on p53 pulses may impact on cell fate decisions, as p53 oscillations favor resumed proliferation rather than cell cycle exit (Purvis et al., 2012).

## CONCLUSION

**FIGURE 1:**
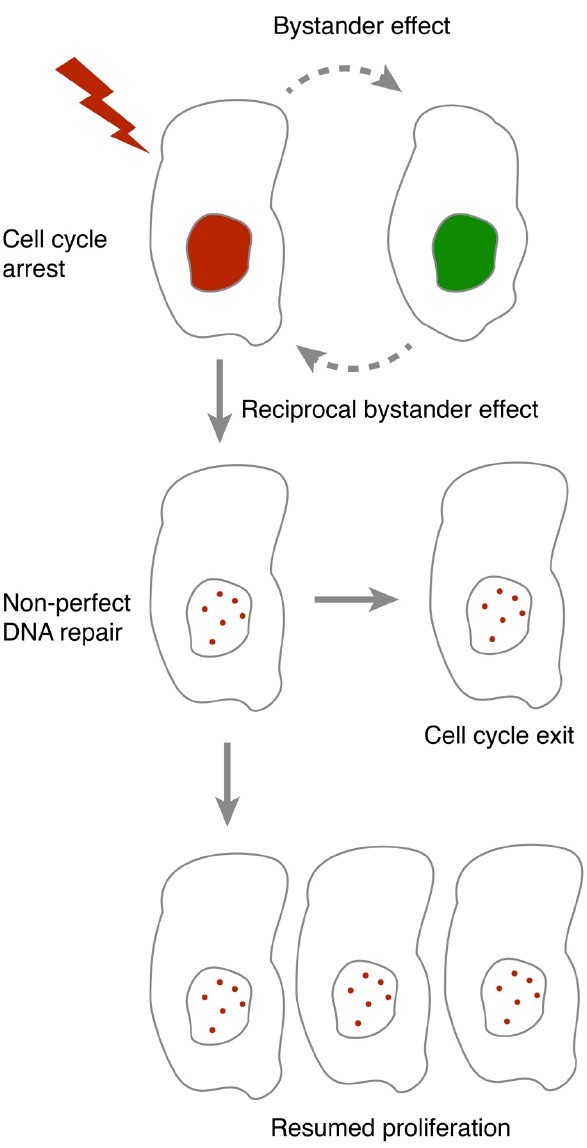
**In the presence of DNA damage, cells either pause or terminally exit the cell cycle.** As DNA repair and cell cycle checkpoint maintenance are not always accurate, resumption of proliferation after a cell cycle pause may lead to propagation of mutations. Bystander communication forms an intercellular feedback that may contribute to whether resumption of proliferation may occur.

As both DNA repair and cell cycle checkpoint maintenance is not perfect, the occurrence of DNA damage to a cell constitutes a risk for establishment and propagation of genomic changes. By forcing a cell to permanently withdraw from the cell cycle, the risk associated with such changes can be reduced. Indeed, a permanent cell cycle exit is suggested to function as a tumor barrier after oncogene-induced DNA damage in S phase (Bartkova et al., 2006; Di Micco et al., 2006), a phase that may be particularly susceptible for RIBE (Burdak-Rothkamm et al., 2007). However, the determinants for when cells exit the cell cycle are not clear. Interestingly, p53, the key regulator of cell cycle exit may both modulate and respond to bystander communication. This opens up for the possibility that feedback within a population impacts on whether cell cycle exit occurs (Figure 1).

The original definition of a checkpoint is a mechanism that is checking to see that the prerequisites (for a process as cell cycle progression) have been properly satisfied (Hartwell and Weinert, 1989). A growing body of evidence suggests that upon damage a cell changes its microenvironment and spreads a signal to neighboring cells to communicate that damage is inflicted. Whether the spread of a signal from a damaged cell is a call for help or a warning is still not clear. The spread is likely to contribute to an effective population response and to assist to eliminate severely damaged cells. However, how cell intrinsic and cell extrinsic pathways interact to determine the fate of a damaged cell remains unclear. Nonetheless, the existence of cell–cell communication affecting DDR pathways calls for caution in evaluating experiments without controlling the local environment, as factors as cell confluence may impact on experimental outcome.

## OUTLOOK

The bystander response, as a cause of genome instability, is implicated in induction of mutations leading to secondary cancers (Coates et al., 2008; Lorimore et al., 2008; Mancuso et al., 2008). In contrast to partial or total gene deletion in directly irradiated cells, bystander cells show primarily point mutations (Huo et al., 2001). Thus, surrounding cells may receive a more subtle genomic change that may promote survival. Early tumor development is accompanied by DNA damage also in the absence of treatment, where activation of a DDR can precede p53 mutations and defects in DNA damage signaling (Bartkova et al., 2005; Gorgoulis et al., 2005). Whether a bystander effect may contribute to increase malignant transformation during tumorigenesis remains to be studied. However, it is possible that a group of early tumor cells may not only collectively enhance the amount of DNA damage per cell, but may also impact on whether proliferation will be resumed. Due to the non-perfect DNA repair and checkpoint maintenance, such resumed proliferation may increase the risk for malignant transformation.

p53 and its associated pathways are altered in more than half of all human cancers, likely reflecting the importance of p53 for cellular fate. It is tempting to speculate that alteration in the p53 pathway can give flexibility to a cell to respond to different extrinsic signals and to better adapt to the environment. Understanding how the bystander effect couples to cell fate decision may impact on risk assessment and indicate novel targets to increase the efficiency of chemo- and radiation therapy.

### Conflict of Interest Statement

The authors declare that the research was conducted in the absence of any commercial or financial relationships that could be construed as a potential conflict of interest.
